# Influence of Electrochemical Oxidation in H_2_SO_4_ and H_3_PO_4_ on the Electrochemical Behavior of Ti-6Al-4V ELI Alloy in Artificial Biological Media Mimicking Physiological and Pathological Environments

**DOI:** 10.3390/ma19081530

**Published:** 2026-04-10

**Authors:** Lidia Benea, Nicoleta Bogatu, Veaceslav Neaga, Elena Roxana Axente

**Affiliations:** 1 Competences Centre—Interfaces-Tribocorrosion-Electrochemical Systems (CC-ITES), “Dunarea de Jos” University of Galati, 47 Domneasca Street, 800008 Galati, Romania; veaceslav.neaga@ugal.ro; 2 Interdisciplinary Research Centre in the Field of Eco-Nano Technology and Advance Materials CC-ITI, “Dunarea de Jos” University of Galati, 47 Domneasca Street, 800008 Galati, Romania; 3 Center for Research and Technology Transfer in the Medico-Pharmaceutical Field, “Dunarea de Jos” University of Galati, 800008 Galati, Romania; elena.axente@ugal.ro; 4 Department of Pharmaceutical Sciences, Faculty of Medicine and Pharmacy, “Dunarea de Jos” University of Galati, 35, Al. I. Cuza Street, 800216 Galati, Romania

**Keywords:** Ti-6Al-4V ELI alloy, anodic oxidation, nanoporous oxide layer, corrosion resistance, Ringer solution, Ringer solution + 40 g/L H_2_O_2_

## Abstract

This research investigates the effects of electrochemical oxidation on surface properties and corrosion performance of the Ti-6Al-4V ELI alloy intended for biomedical applications. Electrochemical anodization is performed in 1 M H_2_SO_4_ and 1 M H_3_PO_4_ electrolytes at applied potentials of 200, 250, and 275 V for 1 min. Morphological characteristics and chemical constitution of the oxide films are investigated by SEM-EDS analysis, while surface roughness, wettability, and microhardness are evaluated using profilometry, contact angle measurements, and Vickers microhardness testing. Electrochemical behavior is assessed by monitoring free potential (OCP) and electrochemical impedance spectroscopy in Ringer solution and Ringer solution containing 40 g/L hydrogen peroxide. Among the investigated conditions, anodization at 200 V for 1 min provides the most favorable surface morphology, producing well-defined and uniformly distributed nanopores while maintaining the structural stability of the oxide layer. Oxidation in 1 M H_2_SO_4_ leads to a more homogeneous nanoporous structure, higher surface roughness, improved hydrophilicity, and increased microhardness compared to 1 M H_3_PO_4_ treatment. Electrochemical impedance spectroscopy analysis reveals superior corrosion resistance for all oxidized samples in comparison with the untreated alloy. The oxide layers obtained in sulfuric acid exhibit the highest polarization resistance and electrochemical stability in simulated physiological environments.

## 1. Introduction

Electrochemical oxidation represents one of the most accessible and efficient methods for modifying the surface of metallic materials, being recognized for its low cost, technological simplicity, and the possibility of fine control over the properties of the formed layer [1,2,3,4]. Compared to other surface treatment techniques, electrochemical oxidation enables the formation of uniform, adherent, and stable protective layers using relatively simple equipment and readily available electrolytes [1,3,5]. For these reasons, the method is extensively applied in both industry and advanced materials research [6].

In the case of titanium-based alloys, electrochemical oxidation plays a particularly important role due to titanium’s natural ability to form a passive TiO_2_ film [1,2,7]. The properties of this layer, including thickness, morphology, composition, and crystalline structure, can be tailored through the electrochemical conditions and the characteristics of the electrolyte. Consequently, surfaces with improved corrosion resistance, high electrochemical stability, and, in certain applications, enhanced biocompatibility can be obtained [1,2,7].

The Ti-6Al-4V ELI alloy (Titanium Grade 23) is a low-interstitial variant of Ti-6Al-4V, widely used in the biomedical field [8]. The reduced oxygen and iron content provide superior ductility and fracture toughness compared to Grade 5 while maintaining high mechanical strength [9,10]. In biomedical applications, the electrochemical behavior of this alloy is essential, as corrosion resistance is strongly dependent on passive film stability in physiological environments and, consequently, on the long-term safety of implants [5].

Corrosion is a complex electrochemical phenomenon responsible for the progressive degradation of metallic materials in contact with the surrounding environment. In the biomedical field, corrosion may induce the release of metallic ions, inflammatory reactions, and reduced duration of implantable devices [11]. Although titanium is considered highly corrosion resistant due to spontaneous passivation [5,7,12], its performance critically depends on the integrity and stability of the oxide layer [6,11]. In this context, controlled electrochemical treatments can improve the protective properties of the surface [13].

Numerous studies from the literature have investigated the influence of electrochemical oxidation in different electrolytes on titanium alloys, highlighting the significant effect of acid type on the structure and properties of the oxide layer [1,2,3,7,12,13,14,15,16,17,18,19,20,21,22,23,24,25,26,27,28]. Anodic oxidation is a complex process of modifying anodic layers on titanium, involving the adjustment of electrochemical variables, including electrolyte composition and concentration, applied potential/current, temperature, and other relevant factors [29]. Alipal J. et al. [30], in a retrospective review of the evolution of electrochemical oxidation of titanium implants, emphasized that in acidic electrolytes (acetic acid, sulfuric acid, hydrochloric acid, and phosphoric acid) and/or neutral electrolytes (sodium sulfate), various microporous TiO_2_ anodic layer patterns with specific crystalline structures can be obtained. In contrast, alkaline electrolytes (sodium hydroxide and potassium hydroxide) produce amorphous nanoporous TiO_2_ anodic layers. It is also known that different electrolytes exhibit varying ionic conductivities within an electrochemical cell, with acidic and neutral electrolytes providing higher electrical conductivity than alkaline electrolytes [30].

This leads to a higher driving force for electric arc formation and dielectric breakdown, resulting in an increased oxidation rate [30].

According to experimental studies, anodic oxidation in a 1 M H_2_SO_4_ solution has proven to be one of the most efficient methods for obtaining commercially pure titanium implants with bioactive and antibacterial properties [31,32]. Furthermore, Yu Mori et al. [33] reviewed comparative studies conducted in CH_3_COOH (weak acid) and H_2_SO_4_ (strong acid) solutions during the electrochemical anodization of TiNbSn alloy. In this context, anatase TiO_2_ crystallization is obtained in 1 M acetic acid electrolyte, whereas rutile-type TiO_2_ is developed in 1 M sulfuric acid. Two-dimensional profilometry of the anodized surfaces indicated an average thickness of 2.0 μm for TiO_2_ formed in 1 M CH_3_COOH and 2.3 μm for samples immersed in 1 M H_2_SO_4_. TEM analysis shows that the oxide film obtained in 1 M CH_3_COOH had a thickness of approximately 370 nm, whereas the oxide film formed in 1 M H_2_SO_4_ reached approximately 7.7 μm, with a significantly higher proportion of internal pores.

Phosphoric acid (H_3_PO_4_) is widely employed in combination with fluoride-based electrolytes owing to its capacity to form barrier-type TiO_2_ films. With increasing oxide layer thickness, the electrical resistance correspondingly increases. Furthermore, the acidic constituents of the H_3_PO_4_ solution act as a barrier to ion and electron transport, thereby retarding and ultimately inhibiting the oxidation process. The final oxide thickness is limited to a few hundred nanometers due to the presence of a pore-free barrier layer, resulting in compact nanotubular structures [34]. Some researchers have observed that the addition of phosphoric acid to anodizing solutions significantly reduces pore size and length while simultaneously increasing the diameters of nanostructured TiO_2_ in nanotube form. Moreover, when the H_3_PO_4_ concentration exceeds 10% of the total electrolyte volume, nanotubular structures cannot be generated because PO_4_^3−^ anions inhibit the diffusion of F^−^ anions and O^2−^ and/or Ti^4+^ ions, which are necessary for nanotube formation [35,36].

Katunar et al. [37] investigated the effect of anodic oxidation at fixed potentials of 30 and 60 V for 1 h in 1 mol L^−1^ H_3_PO_4_ electrolyte on commercially pure zirconium, aiming to reduce metallic ion release in biological media and enhance osseointegration. Subsequent in vivo tests demonstrated increased local cellular activity on anodized zirconium surfaces. Gomez Sanchez et al. [38] also found that electrochemical anodization in H_3_PO_4_ significantly influences the biocompatibility and corrosion performance of commercially pure zirconium in simulated body fluid (SBF). Thus, sulfuric acid-based electrolytes are known for their ability to generate compact and relatively uniform layers, whereas phosphoric acid-based electrolytes may promote the embedding of phosphate species into the oxide layer, influencing both the electrochemical behavior and the biological interaction of the surface [13,14,26].

However, most existing research focuses either on CP titanium or Ti Grade 5 alloy, while comparative studies specifically dedicated to Titanium Grade 23 remain limited.

The novelty of the present research consists of a comparative analysis of the influence of electrochemical oxidation in 1 M H_3_PO_4_ and 1 M H_2_SO_4_ media on the electrochemical behavior of Ti-6Al-4V ELI alloy by correlating process parameters with oxide layer properties and corrosion performance. Investigating the differences induced by electrolyte composition and their impact on electrochemical stability provides relevant insight for biomedical applications.

## 2. Materials and Methods

### 2.1. Sample Preparation

The experimental investigations are carried out on Ti-6Al-4V ELI (Titanium Grade 23) alloy supplied by Goodfellow (Cambridge, Huntingdon, UK), delivered as rolled plates with nominal dimensions of 30 cm × 30 cm × 0.15 cm. The elemental composition of the Ti Grade 23 (Ti-6Al-4V ELI) alloy used in this study is as follows (wt.%): C—0.03, H—0.003, Fe—0.10, O—0.13, N—0.01, Al—5.5–6.5, V—3.5–4.5, Ti—balance, with other elements up to 0.3 [2].

Test samples are sectioned from the plates into rectangular specimens with dimensions 2.5 cm × 2.5 cm × 0.15 cm. Prior to surface treatment, the samples are subjected to alkaline degreasing in a 50 g/L NaOH solution, followed by acid pickling in diluted hydrochloric acid (HCl, 1:1 *v*/*v*) to eliminate surface contaminants and native oxide residues. After chemical cleaning, the specimens are thoroughly rinsed with distilled water. Each sample is electrically connected to a copper wire and mounted in epoxy resin, leaving an active surface area of 5 ± 0.3 cm^2^ for electrochemical oxidation and electrochemical measurements.

### 2.2. Experimental Procedure

Electrochemical oxidation of the Ti-6Al-4V ELI (Titanium Grade 23) specimens is conducted using a programmable DC power source (TDK Lambda GEN300-8, TDK-Lambda Corporation, Tokyo, Japan) integrated into a conventional two-electrode electrochemical configuration. The experimental setup included an external cooling circuit to ensure temperature stabilization of the electrolyte throughout the anodization process. Acidic electrolytes consisting of 1 M H_3_PO_4_ and 1 M H_2_SO_4_ solutions (350 mL) were prepared using phosphoric acid (85 wt.%) and sulfuric acid (96 wt.%) purchased from Chemical Company S.A., Iași, Romania. The solutions were prepared using Type II distilled water obtained in the laboratory using an AquaMAX™-Basic 361 (YOUNG IN Chromass Co., Ltd., Anyang-si, Republic of Korea) purification system. The titanium alloy sample is the WE (working electrode) with an active surface area of 5 ± 0.3 cm^2^, while a Ti-6Al-4V ELI plate is used as the counter electrode, presenting an effective area of 5 ± 0.2 cm^2^. The near-equal electrode surface ratio is selected to promote homogeneous electric field distribution and uniform oxide growth.

Prior to each anodization run, both electrodes are degreased in ethanol, ultrasonically cleaned for 5 min, thoroughly rinsed with distilled water, and subsequently air-dried.

The oxidation treatments are performed in potentiostatic mode at a controlled temperature of 22 ± 1 °C. To prevent temperature fluctuations, the electrochemical cell was equipped with an external cooling system, ensuring thermal stability of the electrolyte during anodization. Experiments were conducted at applied voltages of 200–275 V with a fixed oxidation duration of 60 s. All experimental trials are conducted under similar operational conditions and repeated five times to verify reproducibility and ensure statistical reliability of the results.

The electrochemical corrosion behavior of the investigated samples is evaluated using a PGZ301 potentiostat/galvanostat system (Radiometer Analytical SAS, Villeurbanne, France) operated with VoltaMaster 4 software (version 7.10). The measurements are carried out in a conventional three-electrode electrochemical cell configuration having a working electrode (the tested samples), a platinum-rhodium grid as counter electrode, and an Ag/AgCl electrode as reference electrode. For electrochemical corrosion evaluation, Ti-6Al-4V ELI samples anodized at 200 V for 1 min in 1 M H_3_PO_4_ and 1 M H_2_SO_4_ were selected as the most suitable within the investigated range, based on the formation of a uniform and well-defined nanoporous structure observed by SEM analysis. This condition provides a balanced morphology with homogeneous pore distribution while avoiding excessive pore growth at higher voltages. Subsequent electrochemical investigations were therefore performed on samples anodized at 200 V to evaluate the corrosion behavior of surfaces exhibiting the most favorable morphological characteristics.

The corrosion tests are performed in 150 mL of simplified Ringer solution. To simulate an aggressive oxidative environment associated with inflammatory processes in vivo, an additional test medium is prepared by adding H_2_O_2_, 30% *w*/*w* analytical grade, to the simplified Ringer solution to obtain a final concentration of 40 g/L. Since the local concentration of reactive oxygen species in vivo is difficult to quantify, elevated H_2_O_2_ levels are commonly used in in vitro studies to accelerate corrosion processes and to better differentiate the performance of surface treatments [2]. In this study, a concentration of 40 g/L (4% *w*/*v*) was selected as a compromise between moderately oxidative and excessively aggressive conditions, enabling a “worst-case” evaluation of surface stability [2]. The composition and physicochemical parameters of the simplified Ringer solution are previously reported in another research paper [1]. The electrochemical experimental protocol included monitoring the open circuit potential (OCP) followed by electrochemical impedance spectroscopy (EIS) measurements performed at free potential. Electrochemical impedance spectra are recorded using sinusoidal perturbation with an AC amplitude of 10 mV over a frequency range from 100 kHz to 10 mHz, with data acquisition performed at 20 s intervals.

The experimental EIS data are fitted using equivalent circuit-based models with ZView software (version 3.4). The integrity of the fitting procedure is assessed by chi-square (χ^2^) values lower than 10^−3^.

All measurements are conducted at 37 ± 1.5 °C. A thermostatic control system is employed to maintain constant electrolyte temperature throughout the electrochemical experiments.

### 2.3. Ex Situ Characterization

The morphological features and elemental composition of the oxide films formed on the Ti-6Al-4V ELI alloy are examined using an FEI QUANTA 200 scanning electron microscope (FEI Company, Hillsboro, OH, USA), coupled with an energy-dispersive X-ray spectroscopy (EDS) system operated through EDAX Genesis 5.10 software. To improve surface conductivity and avoid electrostatic charging during analysis, the samples are sputter-coated with a thin gold layer for approximately 10 s prior to observation.

The surface wettability is assessed through static contact angle measurements using a 5 μL droplet of Ringer solution maintained at 37 ± 1.5 °C, carefully placed on the sample surface. The analyses are carried out with an OCA 15EC contact angle goniometer (DataPhysics Instruments GmbH, Filderstadt, Germany), operated with SCA20 software version 4.3.19. The contact angle values are calculated based on the Young–Laplace fitting method. For each specimen, five independent measurements are conducted, and the final results are expressed as the arithmetic means of the recorded values.

The surface microhardness of the investigated samples is measured using a Leitz microhardness tester (Ernst Leitz GmbH, Wetzlar, Germany). Measurements are performed with a standard Vickers diamond indenter under a load of 200 g (0.2 kgf) applied for a dwell time of 15 s. To obtain reliable and reproducible results, three indentations are carried out on each sample, and the reported values represent the average of the measurements obtained.

The surface roughness (2D) of the samples is evaluated using a portable Surftest SJ-210 Series profilometer (Mitutoyo, Kawasaki, Japan). The stylus is guided along the biomaterial surface with controlled movement, before and after anodic oxidation, over a scanning length of 4 mm and at a traverse speed of 0.25 mm/s. To obtain statistically relevant data, three measurements are performed for each sample. The results are averaged to determine the mean roughness values required for subsequent comparative analysis.

## 3. Results and Discussion

### 3.1. Surface Morphology and Chemical Composition of Ti-6Al-4V ELI Before and After Anodic Oxidation

**Figure 1:**
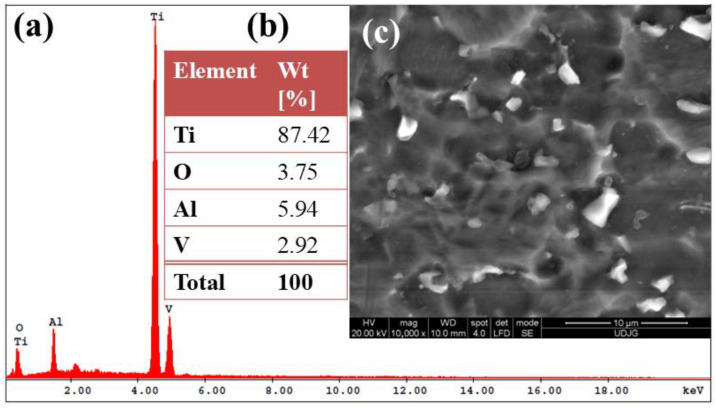
SEM and EDS characterization of the untreated Ti-6Al-4V ELI alloy: (**a**) representative EDS spectrum; (**b**) quantitative elemental composition obtained from EDS analysis; (**c**) SEM image illustrating the surface morphology.

Figure 1a–c presents the SEM and EDS results obtained from the initial morpho-compositional characterization of the untreated Ti-6Al-4V ELI alloy. The EDS spectra (Figure 1a,b) confirm the presence of the main alloying elements specific to Ti-6Al-4V ELI. The quantified mass percentages for the non-oxidized samples are determined as follows: Ti-87.42 ± 0.35%; Al-5.94 ± 0.15%; V-2.92 ± 0.45%; and O-3.75 ± 0.19%.

The estimated TiO_2_ content, derived from the oxygen mass percentage, is approximately 9.36 ± 0.47%, corresponding to the naturally developed passive film. SEM observations reveal a non-uniform surface morphology with irregular topographical features and heterogeneously distributed oxide formations, typical for untreated titanium alloys.

Figure 1c, the surface morphology of the untreated Ti-6Al-4V ELI alloy, reveals a non-uniform, irregular topography. No visible defects associated with mechanical post-processing are observed; however, oxide formations can be identified as opaque, heterogeneously distributed surface features.

**Figure 2:**
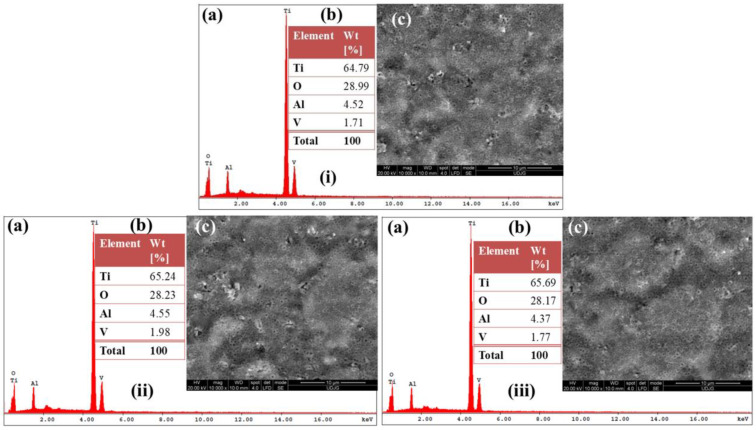
SEM–EDS dataset corresponding to the electrochemically oxidized Ti-6Al-4V ELI alloy samples: (**i**) anodized Ti-6Al-4V ELI alloy at 200 V for 1 min in 1 M H_2_SO_4_; (**ii**) anodized Ti-6Al-4V ELI alloy at 250 V for 1 min in 1 M H_2_SO_4_; (**iii**) anodized Ti-6Al-4V ELI alloy at 275 V for 1 min in 1 M H_2_SO_4_; (**a**) representative EDS spectrum; (**b**) quantitative elemental composition obtained from EDS analysis; (**c**) SEM image illustrating the surface morphology.

Figure 2i–iii show the SEM and EDS characterization of Ti-6Al-4V ELI alloy samples anodically oxidized in 1 M H_2_SO_4_ at different applied potentials of 200 V, 250 V, and 275 V for a duration of 1 min.

The SEM micrographs (Figure 2(ic–iiic)) reveal significant morphological modifications compared to the untreated surface, indicating the growth of a developed oxide film. The anodized surfaces exhibit a more homogeneous and compact structure, with characteristic oxide features distributed uniformly across the substrate. No visible cracks or delamination phenomena are observed, suggesting good adhesion of the anodically formed oxide film.

Based on the oxygen mass percentage obtained from EDS analysis (Figure 2(ib–iiib)) and considering the stoichiometric composition of TiO_2_, the amount of titanium dioxide formed on the surface of samples anodized in sulfuric acid is estimated. The estimated TiO_2_ content on the anodized surfaces is calculated based on the oxygen mass percentage obtained from EDS analysis. The TiO_2_ fraction is determined to be approximately 72.39 ± 0.75% at 200 V, 70.59 ± 0.86% at 250 V, and 70.35 ± 0.49% at 275 V for 1 min of anodization in sulfuric acid. These values demonstrate that anodic oxidation in sulfuric acid leads to the growth of a predominantly TiO_2_-rich surface layer, with only minor variations in oxide content as the applied potential increases.

The slight decrease in the estimated TiO_2_ percentage at higher voltages may be associated with structural rearrangements within the oxide layer or with increased incorporation of electrolyte species.

The EDS and SEM characteristics of the Ti-6Al-4V ELI anodically oxidized in 1 M H_3_PO_4_ at applied potentials of 200 V, 250 V, and 275 V for 1 min are previously reported [2]. For clarity, the corresponding SEM-EDS results are not reproduced in the present study but are referenced for comparative analysis with the samples anodized in sulfuric acid. According to the data reported in [2], the estimated TiO_2_ content formed anodically on Ti-6Al-4V ELI in 1 M H_3_PO_4_ modified surfaces is 91.45 ± 0.14% at 200 V, 93.77 ± 0.29% at 250 V, and 89.10 ± 0.38% at 275 V [2].

**Figure 3:**
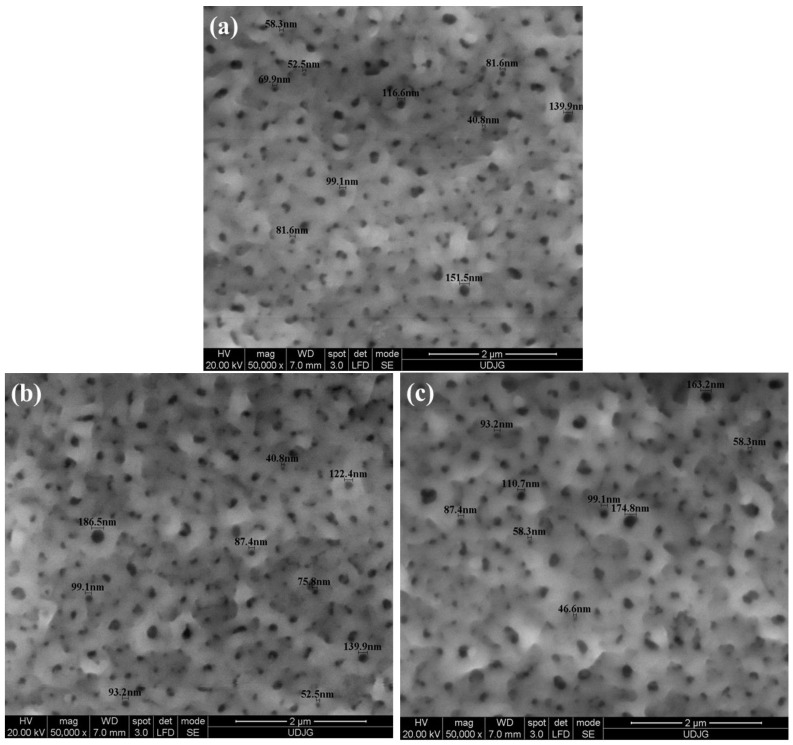
High-magnification SEM images (50,000×) illustrating the nanoporous morphology of Ti-6Al-4V ELI alloy samples anodized in 1 M H_2_SO_4_ at different applied voltages: (**a**) 200 V; (**b**) 250 V; (**c**) 275 V (1 min).

The SEM micrographs in Figure 3 illustrate the surface morphology and nanopore size at a magnification of 50,000×. The images correspond to Ti-6Al-4V ELI alloy samples anodized in 1 M H_2_SO_4_ for 1 min at applied voltages of 200 V, 250 V, and 275 V (Figure 3a–c), highlighting the variations in nanopore size and distribution as a function of the anodization voltage.

Figure 3a–c presents the SEM images of Ti-6Al-4V ELI alloy samples oxidized in 1 M H_2_SO_4_ for 1 min at 200 V, 250 V, and 275 V. The formation of a well-defined nanoporous structure with a relatively uniform pore distribution across the entire surface can be observed. As the applied voltage increases, the surface morphology becomes more evident, and the pore size gradually increases.

The average nanopore diameter determined for the Ti-6Al-4V ELI alloy anodized in 1 M H_2_SO_4_ is 83.54 ± 3.8 nm at 200 V, 99.73 ± 6.9 nm at 250 V, and 104.88 ± 11.6 nm at 275 V, indicating a directly proportional relationship between the anodization voltage and the size of the formed nanopores.

The SEM images of the Ti-6Al-4V ELI alloy samples anodized in 1 M H_3_PO_4_ are previously published [2]. However, the average diameters of the formed nanopores are 98.10 ± 4.6 nm at 200 V, 113.32 ± 10.9 nm at 250 V, and 139.20 ± 13.2 nm at 275 V [2].

The morpho-compositional (SEM-EDS) results obtained for non-oxidized Ti-6Al-4V ELI alloy samples and those anodically oxidized in 1 M H_3_PO_4_ and 1 M H_2_SO_4_ solutions at 200 V, 250 V, and 275 V for 1 min reveal significant differences in surface topography and oxide layer characteristics.

The surface analysis of the non-oxidized Ti-6Al-4V ELI alloy shows non-uniform relief without mechanical defects resulting from the final processing stage. Locally, oxides with asymmetrical, opaque white structures can be observed. After electrochemical oxidation in phosphoric acid, a non-homogeneous film develops on the surface, characterized by alternating regions with pores of varying sizes, including small and large ones, some fully formed and others either partially developed or lacking porosity. This heterogeneity becomes more pronounced as the applied voltage increases from 250 V to 275 V.

In contrast, a mesoporous oxide layer consisting of predominantly round, isolated pores relevant for this study is obtained only for the Ti-6Al-4V ELI alloy samples anodized in sulfuric acid. Comparative analysis of morphological characteristics of oxide films formed in sulfuric and phosphoric acids, depending on anodization potential, reveals that 200 V is optimal for achieving well-defined mesoporous structures via electrochemical anodization in phosphoric or sulfuric acid. The comparative study of the morphology of oxide layers produced in 1 M H_3_PO_4_ and 1 M H_2_SO_4_, depending on the anodization potential, suggests that 200 V is the most appropriate value for generating well-defined mesoporous structures in either electrolyte.

These findings are consistent with previous scientific studies reporting the formation of suitable porous layers in phosphoric acid at anodization potentials ranging from 150 V to 250 V [26,27,39,40,41], while well-defined nanoporous layers have been observed in sulfuric acid at potentials between 150 V and 240 V [26,41,42].

An important advantage of using sulfuric acid lies in the more homogeneous anodically oxidized surface morphology and the higher density of nanopores compared to 1 M H_3_PO_4_, where a more heterogeneous morphology with larger but fewer pores is typically observed. This difference can be attributed to the oxygen-related reactions occurring in the electrolyte during the electrochemical oxidation process; due to its higher acidity compared to H_3_PO_4_, H_2_SO_4_ promotes pore nucleation more effectively [14].

### 3.2. Evaluation of Vickers Microhardness of Ti-6Al-4V ELI Alloy Before and After Anodic Oxidation

**Figure 4:**
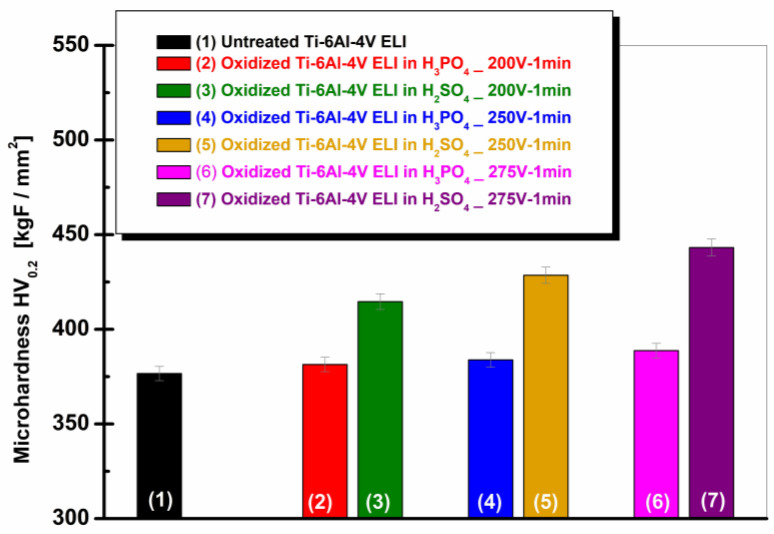
Evolution of Vickers microhardness for the surfaces of untreated and electrochemically oxidized Ti-6Al-4V ELI samples: (1) untreated Ti-6Al-4V ELI; (2) oxidized Ti-6Al-4V ELI (200 V–1 min) in 1 M H_3_PO_4_; (3) oxidized Ti-6Al-4V ELI (200 V–1 min) in 1 M H_2_SO_4_; (4) oxidized Ti-6Al-4V ELI (250 V–1 min) in 1 M H_3_PO_4_; (5) oxidized Ti-6Al-4V ELI (250 V–1 min) in 1 M H_2_SO_4_; (6) oxidized Ti-6Al-4V ELI (275 V–1 min) in 1 M H_3_PO_4_; (7) oxidized Ti-6Al-4V ELI (275 V–1 min) in 1 M H_2_SO_4_.

Figure 4 shows the evolution of Vickers microhardness (HV0.2) for the untreated and electrochemically anodized Ti-6Al-4V ELI alloy samples in 1 M H_3_PO_4_ and 1 M H_2_SO_4_ at various applied potentials.

From Figure 4, the non-oxidized Ti-6Al-4V ELI alloy exhibits a microhardness value of 376.68 ± 3.76 HV, which serves as the reference. After anodization in 1 M H_3_PO_4_, the microhardness increases slightly with increasing voltage, reaching 381.43 ± 3.81 HV at 200 V, 383.84 ± 2.62 HV at 250 V, and 388.73 ± 3.88 HV at 275 V. This indicates a modest improvement in surface hardness compared to the untreated condition.

In contrast, the samples anodized in 1 M H_2_SO_4_ show a significantly higher increase in microhardness. The values increased to 414.64 ± 4.14 HV at 200 V, 428.57 ± 4.32 HV at 250 V, and 443.22 ± 4.51 HV at 275 V. The increase is more pronounced with increasing anodization potential, suggesting the development of a harder and more compact oxide film.

Anodization process leads to an enhancement of surface microhardness for all tested conditions, however, sulfuric acid produces a substantially greater hardening effect than phosphoric acid. The highest microhardness value, 443.22 ± 4.51 HV, is obtained for the sample anodized in 1 M H_2_SO_4_ at 275 V for 1 min, confirming the strong influence of both electrolyte type and applied voltage on the surface mechanical properties of the Ti-6Al-4V ELI alloy.

### 3.3. Roughness of Ti-6Al-4V ELI Alloy Before and After Anodic Oxidation

**Figure 5:**
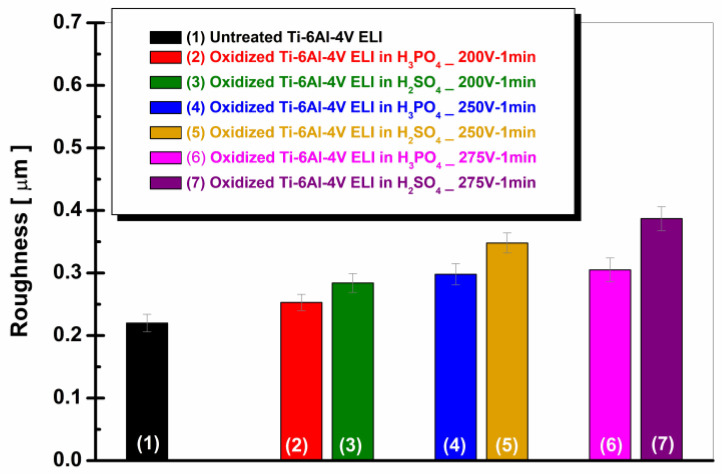
Evolution of roughness for the surfaces of untreated and electrochemically oxidized Ti-6Al-4V ELI samples: (1) untreated Ti-6Al-4V ELI; (2) oxidized Ti-6Al-4V ELI (200 V–1 min) in 1 M H_3_PO_4_; (3) oxidized Ti-6Al-4V ELI (200 V–1 min) in 1 M H_2_SO_4_; (4) oxidized Ti-6Al-4V ELI (250 V–1 min) in 1 M H_3_PO_4_; (5) oxidized Ti-6Al-4V ELI (250 V–1 min) in 1 M H_2_SO_4_; (6) oxidized Ti-6Al-4V ELI (275 V–1 min) in 1 M H_3_PO_4_; (7) oxidized Ti-6Al-4V ELI (275 V–1 min) in 1 M H_2_SO_4_.

Figure 5 shows the evolution of surface roughness (Ra, µm) for untreated and electrochemically oxidized Ti-6Al-4V ELI samples in 1 M H_3_PO_4_ and 1 M H_2_SO_4_ over the voltage range of 200–275 V.

From Figure 5, the untreated alloy presents a roughness of 0.220 ± 0.014 µm. Following anodization, the roughness increases under all experimental conditions, indicating significant surface modification induced by the electrochemical oxidation process.

For the samples treated in H_3_PO_4_, the roughness increases gradually with voltage, reaching 0.253 ± 0.013 µm (200 V), 0.298 ± 0.017 µm (250 V), and 0.305 ± 0.029 µm (275 V), suggesting a moderate development of the oxide layer.

In contrast, anodization in H_2_SO_4_ results in a more pronounced increase in roughness: 0.284 ± 0.015 µm (200 V), 0.348 ± 0.016 µm (250 V), and 0.387 ± 0.019 µm (275 V). The increase in roughness at higher voltages reflects a more intense surface structuring process, consistent with enhanced pore formation and oxide growth.

These roughness results are consistent with the SEM-EDS morphological observations, which revealed a more homogeneous and densely nanoporous structure for the samples anodized in H_2_SO_4_ compared to those treated in H_3_PO_4_. The higher roughness values measured for sulfuric acid-treated samples correlate with the more developed porous morphology identified by SEM analysis, confirming the strong influence of electrolyte type and anodization potential on surface characteristics. Similar trends have been reported in the literature, with an increase in surface roughness and pore development observed at higher anodization voltages, particularly in sulfuric acid electrolytes, confirming the strong influence of electrolyte type and applied potential on the surface morphology of titanium alloys [40,41,43].

### 3.4. Wettability of Ti-6Al-4V ELI Alloy Before and After Anodic Oxidation

**Figure 6:**
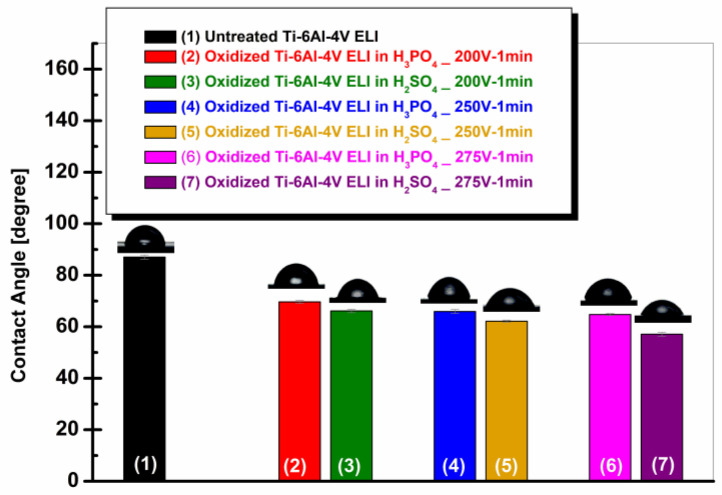
Evolution of contact angle for the surfaces of untreated and electrochemically oxidized Ti-6Al-4V ELI samples: (1) untreated Ti-6Al-4V ELI; (2) oxidized Ti-6Al-4V ELI (200 V–1 min) in 1 M H_3_PO_4_; (3) oxidized Ti-6Al-4V ELI (200 V–1 min) in 1 M H_2_SO_4_; (4) oxidized Ti-6Al-4V ELI (250 V–1 min) in 1 M H_3_PO_4_; (5) oxidized Ti-6Al-4V ELI (250 V–1 min) in 1 M H_2_SO_4_; (6) oxidized Ti-6Al-4V ELI (275 V–1 min) in 1 M H_3_PO_4_; (7) oxidized Ti-6Al-4V ELI (275 V–1 min) in 1 M H_2_SO_4_.

Figure 6 depicts the evolution of average contact angle values for the untreated and electrochemically oxidized Ti-6Al-4V ELI samples in 1 M H_3_PO_4_ and 1 M H_2_SO_4_ at different anodization voltages (200 V, 250 V, and 275 V).

From Figure 6, it can be observed that the non-oxidized titanium sample displays a contact angle of 87.08 ± 0.82°, indicating a slightly hydrophilic surface, situated at the boundary between hydrophobic and hydrophilic behavior. After anodic oxidation, all samples show a significant decrease in contact angle, with values below 70°, demonstrating a clear increase in surface hydrophilicity.

In the 1 M H_3_PO_4_ solution, the contact angle values progressively decrease with increasing applied voltage, from 69.67 ± 0.53° at 200 V to 65.93 ± 0.75° at 250 V and 64.76 ± 0.38° at 275 V [2]. This trend indicates that intensifying the oxidation regime promotes the development of an oxide film with higher surface energy, capable of improving wettability.

For the sample oxidized in 1 M H_2_SO_4_ solution, a more significant decrease in contact angle values is observed, decreasing from 66.15 ± 0.69° at 200 V to 62.17 ± 0.45° at 250 V and 57.11 ± 0.76° at 275 V. Compared to the samples treated in 1 M H_3_PO_4_, systematically lower contact angle values are observed for the titanium alloy samples oxidized in 1 M H_2_SO_4_ at the same applied voltage, with differences of approximately 3.5° at 200 V, 3.8° at 250 V, and 7.6° at 275 V. The difference becomes more pronounced at higher voltages, suggesting a cumulative effect of the electrochemical parameters on the structure and composition of the oxide layer.

These results indicate that anodic oxidation in sulfuric acid leads to a more pronounced increase in the hydrophilic character of the surface compared to phosphoric acid. The reduction in contact angle values is in good agreement with the surface roughness data presented in Figure 5. C.L. Chu and co-workers indicate that wettability, blood compatibility, and thromboresistance can also be improved through anodic oxidation in an H_2_SO_4_ electrolyte [44].

### 3.5. Comparative Corrosion Performance of Untreated and Electrochemically Treated Ti-6Al-4V ELI Alloy in Artificial Biological Media Mimicking Physiological and Pathological Environments

#### 3.5.1. Open Circuit Potential (OCP)

The electrochemical test used to determine the free potential vs. time reflects the potential difference between the working and reference electrodes in each electrolyte under zero-current conditions [45]. Monitoring the OCP evolution enables the assessment of the stability and integrity of protective layers (in this case, the oxide layer), which act as barriers against corrosion processes. Electron transfer through anticorrosive layers or their degradation generally results in a shift in the potential toward more negative values, associated with the exposure of the metallic substrate [45].

According to the literature, more negative OCP values may indicate degradation or dissolution of the passive film and the subsequent onset of corrosion at the substrate level, whereas a shift toward more positive potential is typically correlated with the growth of a stable protective film [45].

However, the corrosion potential should not be interpreted in isolation as a direct indicator of corrosion rate, since more noble (more positive) potential values do not necessarily imply a lower corrosion rate [46]. A proper evaluation of electrochemical behavior requires correlating OCP measurements with other complementary electrochemical techniques [45].

**Figure 7:**
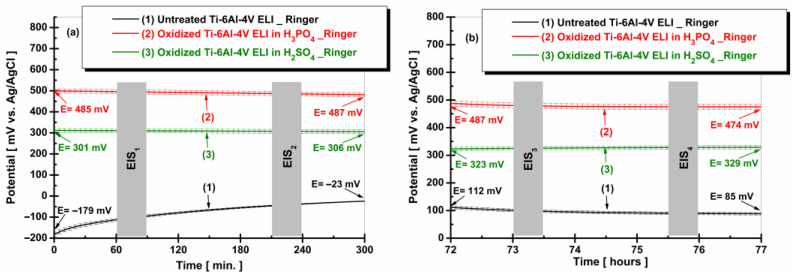
Comparative plots of free potential evolution for non-oxidized Ti-6Al-4V ELI alloy samples and oxidized in phosphoric or sulfuric acid, after immersion in Ringer solution, at two distinct time intervals: (**a**) t_1_—5 h and (**b**) t_2_—77 h.

Figure 7 shows the evolution of free potential for the non-oxidized Ti-6Al-4V ELI alloy sample and oxidized in phosphoric or sulfuric acid, following immersion in Ringer solution, evaluated at two distinct time intervals: (a) t_1_—5 h and (b) t_2_—77 h.

From Figure 7a, it can be observed that the untreated Ti-6Al-4V ELI alloy sample (curve 1) exhibits a sharp increase in the OCP value during the first 300 min of monitoring, without reaching a steady-state condition. The sample starts at an E = −179 ± 19 mV vs. Ag/AgCl upon immersion and reaches −23 ± 4 mV vs. Ag/AgCl at the end of the measurement. The shift toward positive potential values indicates the rapid growth of a native protective oxide film on the material surface; however, this layer dissolves as rapidly as it forms [46].

In the case of curve 2, corresponding to the Ti-6Al-4V ELI sample electrochemically oxidized in H_3_PO_4_ solution, a slight shift in the free potential toward more positive values is noted, the final value after 300 min being 2 ± 0.4 mV higher than the initial value at immersion.

Similarly, the sample treated in sulfuric acid (curve 3) shows a slight shift toward more positive values, with the potential measured at the end of the 300 min being 5 ± 0.9 mV higher than the value recorded at immersion.

From Figure 7b, after three days of immersion, the untreated sample exhibits a shift in the potential toward negative values. The sample electrochemically oxidized in H_2_SO_4_ shows a shift toward more positive values. Comparing Figure 7a, curve 3, with Figure 7b, curve 3, it can be observed that the free potential increased with increasing immersion time. In contrast, the sample treated with H_3_PO_4_ showed a decrease in OCP values as the immersion time increased, indicating a progressive dissolution of the oxide film.

**Figure 8:**
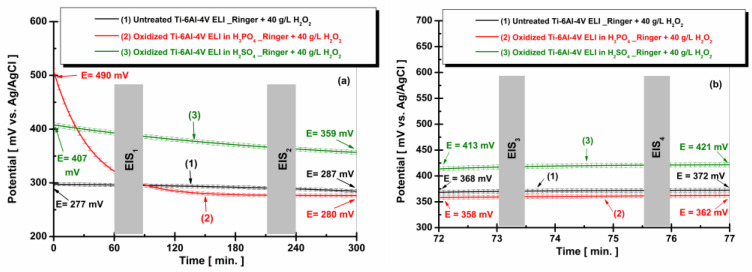
Evolution of the free potential for non-oxidized Ti-6Al-4V ELI samples and those oxidized in phosphoric acid and sulfuric acid after immersion in Ringer solution containing 40 g/L H_2_O_2_ at two time intervals: (**a**) t_1_—5 h and (**b**) t_2_—77 h.

Figure 8 presents the evolution of the free potential for non-oxidized Ti-6Al-4V ELI samples and oxidized samples in phosphoric acid and sulfuric acid after immersion in Ringer solution containing 40 g/L H_2_O_2_ at two time intervals: (a) t_1_—5 h and (b) t_2_—77 h.

By comparing Figure 7a and Figure 8a, it can be observed that the initial OCP values in the medium containing 40 g/L H_2_O_2_ are more positive for the electrochemically oxidized samples than in the simple Ringer solution. This behavior can be attributed to the strong oxidizing character of hydrogen peroxide, which may initially promote the formation or stabilization of a more noble oxide film [46].

However, with increasing immersion time (Figure 7b and Figure 8b), the OCP values tend to stabilize, and the differences between the environments become less pronounced. In the presence of H_2_O_2_, the potentials shift toward slightly lower or stabilized values, suggesting that after the initial oxidation stage, the oxide layer reaches a quasi-steady state. This indicates that prolonged exposure leads to the stabilization of the passive film, while the aggressive oxidative environment may also induce partial modification or restructuring of the oxide layer.

#### 3.5.2. Electrochemical Impedance Spectroscopy (EIS)

EIS is considered one of the most reliable electrochemical techniques for investigating corrosion mechanisms, as it provides detailed information on interfacial processes, charge transfer resistance, and the protective properties of surface films without significantly perturbing the system [45,46].

Considering the complex interface generated by the native oxide layer that forms instantaneously on titanium alloys, this interface mainly consists of the boundary between titanium oxide (TiO_2_) and the saline solution, as well as the interface between the titanium substrate and the titanium oxide film in contact with the testing electrolyte. The titanium oxide film passivates the alloy surface, thereby slowing down the corrosion process. The dissolution of the alloy beneath the oxide layer occurs at a significantly lower rate due to the presence of this passive film. In practice, the passive layer is slowly removed by the corrosive environment but is immediately reformed from the untreated alloy surface.

Therefore, to achieve an accurate fitting of the impedance data, the selection of the equivalent electrical circuits was based on the number of time constants observed in the impedance spectra, as well as on the physical interpretation of the electrochemical processes occurring at the oxide/electrolyte interface.

**Figure 9:**
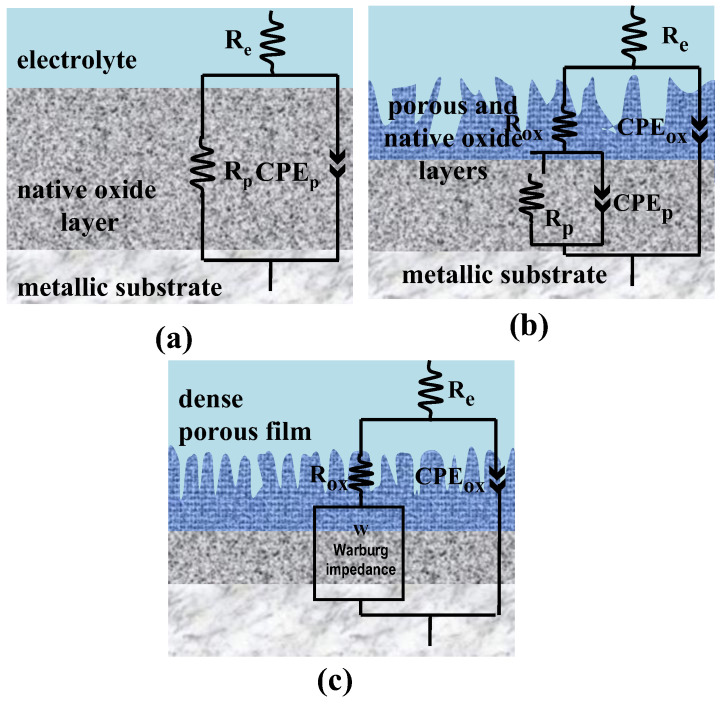
Equivalent electrical circuits used for fitting the EIS plots after corrosion in the studied electrolytes: (**a**) equivalent electrical circuit for non-oxidized Ti-6Al-4V ELI; (**b**) equivalent electrical circuit for Ti-6Al-4V ELI electrochemically oxidized in 1 M H_3_PO_4_; (**c**) equivalent electrical circuit for Ti-6Al-4V ELI electrochemically oxidized in 1 M H_2_SO_4_.

The untreated Ti-6Al-4V ELI alloy exhibits a single time constant associated with the naturally formed passive TiO_2_ layer and can be adequately described using a simple equivalent circuit (Figure 9a).

For the samples anodically oxidized in phosphoric acid, the impedance response reveals two distinct time constants, indicating the formation of a duplex oxide structure consisting of an outer porous layer and an inner compact barrier layer. Accordingly, an equivalent circuit including two resistive and two constant phase elements is employed (Figure 9b).

For the samples oxidized in sulfuric acid, the impedance response shows characteristics of diffusion-controlled processes. This behavior is supported by α values close to 0.5 (Table 1 and Table 2) and is attributed to restricted ionic transport through the nanoporous oxide layer. Consequently, the equivalent circuit includes a Warburg impedance element, replacing the resistive and capacitive elements associated with the interface near the metallic substrate (Figure 9c).

**Figure 10:**
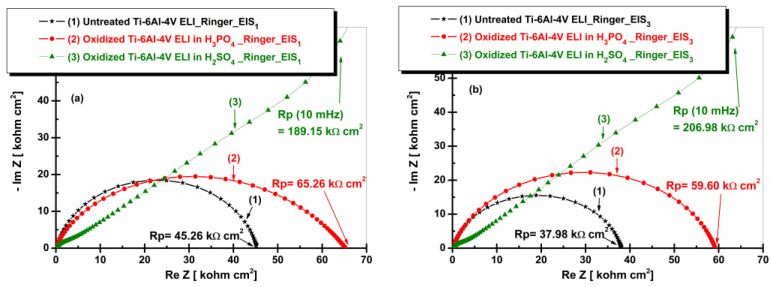
Nyquist plots (EIS) of untreated and anodized Ti-6Al-4V ELI samples in phosphoric and sulfuric acid after immersion in artificial Ringer solution: (**a**) t_1_—after 5 h and (**b**) t_2_—after 77 h.

Figure 10 presents the Nyquist plots for the Ti-6Al-4V ELI alloy in an untreated condition and after electrochemical oxidation after immersion in artificial Ringer solution.

Figure 10a,b, shows the highest value of the polarization resistance (Rp) for the Ti-6Al-4V ELI sample electrochemically oxidized in sulfuric acid solution, while the lowest polarization resistance corresponds to the untreated alloy.

It can also be noted that increasing immersion time leads to an increase in polarization resistance for the electrochemically oxidized Ti-6Al-4V ELI alloy in 1 M H_2_SO_4_, whereas for the untreated alloy and Ti-6Al-4V ELI alloys oxidized in 1 M H_3_PO_4_, the Rp value decreases with increasing immersion time. This behavior suggests that sulfuric acid promotes the formation of a denser and more adherent oxide film, which more effectively restricts the transport of ions and electrons than the oxide film formed on the surface of the Ti-6Al-4V ELI alloys oxidized in 1 M H_3_PO_4_ [26].

**Figure 11:**
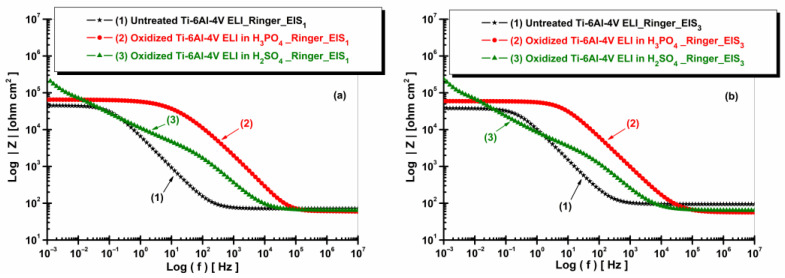
Bode plots (EIS) of impedance modulus vs. log (f) of untreated and anodized Ti-6Al-4V ELI samples in phosphoric and sulfuric acid after immersion in artificial Ringer solution: (**a**) t_1_—after 5 h and (**b**) t_2_—after 77 h.

**Figure 12:**
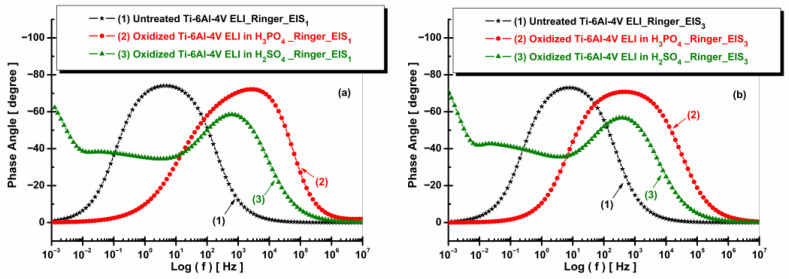
Bode plots (EIS) of phase angle vs. log (f) of untreated and anodized Ti-6Al-4V ELI samples in phosphoric and sulfuric acid after immersion in artificial Ringer solution: (**a**) t_1_—after 5 h and (**b**) t_2_—after 77 h.

Figure 11 and Figure 12 present the EIS plots (Bode representation) showing the impedance modulus vs. logarithm of frequency and the phase angle vs. logarithm of frequency for the non-oxidized and oxidized Ti-6Al-4V ELI after immersion in Ringer solution.

Figure 11 shows that the oxidized Ti-6Al-4V ELI samples exhibit higher impedance modulus values compared to the untreated alloy, confirming the improved corrosion resistance provided by the formed oxide layers.

Figure 12 shows that the untreated Ti-6Al-4V ELI sample exhibits a single-phase angle maximum, indicating the presence of one main time constant associated with the native oxide layer. In contrast, the electrochemically oxidized samples display broader phase angle peaks and a wider frequency range with high phase angle values, suggesting the formation of more complex oxide layers with multiple time constants. The fitted parameters of the equivalent circuit model obtained from the experimental data are provided in Table 1.

**Table 1:** The equivalent circuit parameters obtained by fitting the experimental data for non-oxidized and oxidized Ti-6Al-4V ELI samples in 1 M H_3_PO_4_ and 1 M H_2_SO_4_ after immersion in Ringer solution.

Parameters of the Equivalent Electrical Circuit /Units	Measurement ^1^	Untreated Ti-6Al-4V ELI	Oxidized Ti-6Al-4V ELI in 1 M H_3_PO_4_ 200 V-1 min	Oxidized Ti-6Al-4V ELI in 1 M H_2_SO_4_ 200 V-1 min
Rs [Ω cm^2^]	EIS_1_	71.45	53.66	64.23
	EIS_3_	94.36	56.31	68.41
CPE-P [F/cm^2^]	EIS_1_	2.948 × 10^−5^	1.040 × 10^−6^	2.532 × 10^−6^
	EIS_3_	1.792 × 10^−5^	7.658 × 10^−7^	4.508 × 10^−6^
α	EIS_1_	0.872	0.912	0.804
	EIS_3_	0.873	0.822	0.784
Rp [MΩ cm^2^]	EIS_1_	45.263	0.00826	2.969
	EIS_3_	37.977	3.140	3.040
CPE-Tox [F/cm^2^]	EIS_1_	-	6.382 × 10^−8^	-
	EIS_3_	-	2.994 × 10^−9^	-
α	EIS_1_	-	0.969	-
	EIS_3_	-	0.995	-
Rp [MΩ cm^2^]	EIS_1_	-	65.254	-
	EIS_3_	-	56.025	-
Wo-R [kΩ cm^2^]	EIS_1_	-	-	186.180
	EIS_3_	-	-	203.940
Wo-Tox [F/cm^2^]	EIS_1_	-	-	128.6
	EIS_3_	-	-	144.3
Wo-α	EIS_1_	-	-	0.441
	EIS_3_	-	-	0.488

^1^ Electrochemical impedance spectroscopy (EIS) measurements were performed after different immersion times—EIS_1_ after 5 h and EIS_3_ after 77 h—in order to evaluate the time-dependent corrosion behavior.

From Table 1, it can be observed that the untreated Ti-6Al-4V ELI alloy shows relatively moderate polarization resistance values, suggesting the presence of a naturally formed oxide film with limited protective properties.

In contrast, the electrochemically oxidized samples exhibit modified impedance parameters, indicating the development of more stable and protective oxide layers. For the sample oxidized in 1 M H_3_PO_4_, the value of additional elements (CPE-Tox, α, and Rp) suggests the development of a more complex oxide structure composed of multiple layers. The relatively high Rp values confirm an enhanced corrosion resistance as compared with the untreated alloy. For the sample oxidized in 1 M H_2_SO_4_, the impedance response also includes a Warburg element indicating diffusion-controlled processes occurring through the oxide layer. The high resistance values associated with this element suggest that the formed oxide layer significantly limits ionic transport, contributing to enhanced corrosion protection. Additionally, the constant phase element parameters and their associated α values indicate non-ideal capacitive behavior, which is typically associated with surface heterogeneity or roughness of the oxide films. Oxidation in 1 M H_2_SO_4_ proved to be the most effective, followed by oxidation in 1 M H_3_PO_4_, with both treatments demonstrating significantly better protection than that observed for the non-oxidized samples. These results suggest that the treatment duration significantly influences the development of the protective oxide film and, consequently, the electrochemical protection of the alloy, which is essential for its application in biological environments or in other fields requiring corrosion resistance.

**Figure 13:**
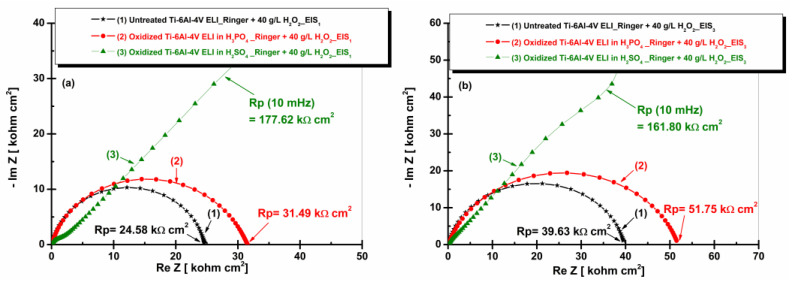
Nyquist plots (EIS) of untreated and anodized Ti-6Al-4V ELI samples in phosphoric and sulfuric acid after immersion in artificial Ringer solution + 40 g/L H_2_O_2_: (**a**) t_1_—after 5 h and (**b**) t_2_—after 77 h.

Figure 13 presents the Nyquist plots obtained for the non-oxidized and oxidized Ti-6Al-4V ELI after immersion in artificial Ringer solution + 40 g/L H_2_O_2_.

By comparing Figure 10 and Figure 13, it can be observed that the samples immersed in Ringer solution + 40 g/L H_2_O_2_ exhibit lower Rp values as compared with samples immersed in solutions without hydrogen peroxide for both investigated immersion times.

This response can be attributed to the more aggressive and oxidizing nature of the medium in the presence of hydrogen peroxide, which promotes electrochemical reactions and may destabilize the protective oxide film developed on the alloy surface [46]. Nevertheless, the samples electrochemically oxidized in 1 M H_2_SO_4_ and 1 M H_3_PO_4_ still maintain higher Rp values as compared to the non-oxidized sample. Among the treated samples, the alloy oxidized in sulfuric acid exhibits superior Rp values as compared with the untreated sample and the sample oxidized in phosphoric acid. Furthermore, increasing the immersion time leads to a decrease in the polarization resistance values, suggesting a gradual reduction in the protective properties of the oxide layer during prolonged exposure to the corrosive environment.

**Figure 14:**
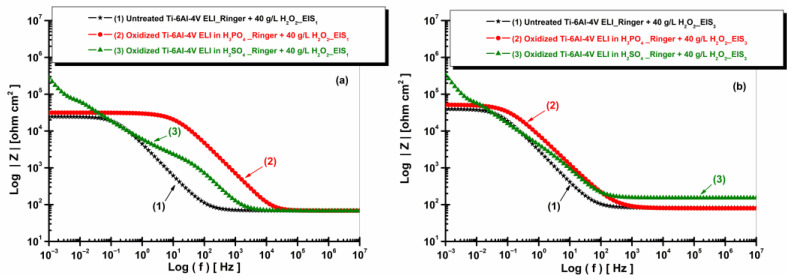
Bode plots (EIS) of impedance modulus vs. log (f) of untreated and anodized Ti-6Al-4V ELI samples in phosphoric and sulfuric acid after immersion in artificial Ringer solution + 40 g/L H_2_O_2_: (**a**) t_1_—after 5 h and (**b**) t_2_—after 77 h.

Figure 14 presents the EIS diagrams in Bode representation, showing the impedance modulus vs. the logarithm of frequency for the non-oxidized and oxidized Ti-6Al-4V ELI samples after immersion in Ringer solution + 40 g/L H_2_O_2_.

**Figure 15:**
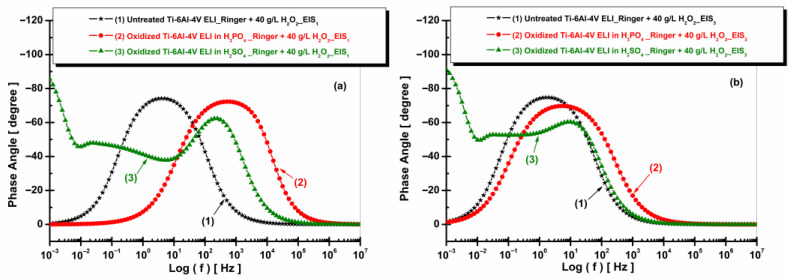
Bode plots (EIS) of phase angle vs. log (f) of untreated and anodized Ti-6Al-4V ELI samples in phosphoric and sulfuric acid after immersion in artificial Ringer solution + 40 g/L H_2_O_2_: (**a**) t_1_—after 5 h and (**b**) t_2_—after 77 h.

Figure 15 presents the EIS diagrams in Bode representation, showing the phase angle vs. logarithm of frequency for the Ti-6Al-4V ELI samples, untreated and electrochemically oxidized, after immersion in artificial Ringer solution + 40 g/L H_2_O_2_.

Table 2 presents the parameters of the equivalent circuit obtained from fitting the experimental data for the Ti-6Al-4V ELI samples, untreated and electrochemically oxidized in 1 M H_3_PO_4_ and 1 M H_2_SO_4_, after immersion in Ringer solution + 40 g/L H_2_O_2_.

**Table 2:** The equivalent circuit parameters obtained by fitting the experimental data for non-oxidized and oxidized Ti-6Al-4V ELI samples in 1 M H_3_PO_4_ and 1 M H_2_SO_4_ after immersion in Ringer solution + 40 g/L H_2_O_2_.

Parameters of the Equivalent Electrical Circuit /Units	Measurement ^1^	Untreated Ti-6Al-4V ELI	Oxidized Ti-6Al-4V ELI in 1 M H_3_PO_4_ 200 V-1 min	Oxidized Ti-6Al-4V ELI in 1 M H_2_SO_4_ 200 V-1 min
Rs [Ω cm^2^]	EIS_1_	68.96	68.86	67.89
	EIS_3_	82.24	79.68	152.3
CPE-P [F/cm^2^]	EIS_1_	4.172 × 10^−5^	2.545 × 10^−7^	3.410 × 10^−6^
	EIS_3_	6.493 × 10^−5^	2.625 × 10^−5^	2.619 × 10^−5^
α	EIS_1_	0.890	0.966	0.915
	EIS_3_	0.887	0.825	0.886
Rp [MΩ cm^2^]	EIS_1_	24.575	1.218	1.740
	EIS_3_	36.632	0.525	3.191
CPE-Tox [F/cm^2^]	EIS_1_	-	9.508 × 10^−7^	-
	EIS_3_	-	9.177 × 10^−7^	-
α	EIS_1_	-	0.702	-
	EIS_3_	-	0.704	-
Rp [MΩ cm^2^]	EIS_1_	-	30.276	-
	EIS_3_	-	51.230	-
Wo-R [kΩ cm^2^]	EIS_1_	-	-	175.880
	EIS_3_	-	-	158.610
Wo-Tox [F/cm^2^]	EIS_1_	-	-	103.3
	EIS_3_	-	-	72.9
Wo-α	EIS_1_	-	-	0.544
	EIS_3_	-	-	0.560

^1^ Electrochemical impedance spectroscopy (EIS) measurements were performed after different immersion times—EIS_1_ after 5 h and EIS_3_ after 77 h—in order to evaluate the time-dependent corrosion behavior.

The results indicate that electrochemical oxidation treatments substantially improve the corrosion resistance of the investigated titanium alloy surfaces. Among the applied treatments, oxidation performed in sulfuric acid proved to be the most effective in generating stable and protective passive films, followed by oxidation in phosphoric acid. The samples covered with electrochemically formed oxide layers exhibited higher polarization resistance values, indicating enhanced corrosion resistance in the tested physiological environments. In contrast, the untreated samples showed lower anticorrosion protection, highlighting the essential role of anodic oxidation in optimizing the surface performance of titanium-based biomaterials and improving their stability under aggressive conditions characteristic of post-operative environments.

## 4. Conclusions

The present study demonstrates that electrochemical oxidation significantly modifies the surface characteristics and corrosion behavior of Ti-6Al-4V ELI alloy in simulated physiological environments. Compared to the untreated alloy, anodized samples exhibit a substantial improvement in surface properties, including increased microhardness, enhanced wettability, and improved corrosion resistance.

The untreated alloy is characterized by a thin native TiO_2_ layer, which provides limited protection, as reflected by lower polarization resistance and reduced surface stability. In contrast, anodization in 1 M H_3_PO_4_ leads to the formation of a nanoporous oxide layer with moderate improvements in surface properties. However, SEM observations reveal a relatively heterogeneous morphology, with pore diameters in the range of 98.10–139.20 nm, which may limit the long-term protective performance of the oxide film.

Anodization in 1 M H_2_SO_4_ results in a more uniform and compact nanoporous TiO_2_ structure, with smaller pore diameters (83.54–104.88 nm) and higher pore density. This morphology is associated with superior electrochemical performance, as confirmed by impedance measurements showing higher polarization resistance and improved stability, even in oxidative environments containing H_2_O_2_.

Among the investigated conditions, anodization at 200 V for 1 min is identified as the most suitable regime for obtaining a homogeneous nanoporous structure, ensuring a favorable balance between oxide growth and structural stability.

Despite these promising results, several limitations should be acknowledged. The study is primarily based on surface and electrochemical characterization, without direct phase identification of the oxide layers. In addition, electrochemical testing was performed only for samples anodized at 200 V, which limits direct comparison of corrosion behavior across all anodization voltages.

Future work will focus on detailed structural characterization of the oxide layers, including phase analysis and thickness evaluation, as well as on investigating the influence of anodization parameters on long-term corrosion performance.

The results indicate that electrochemical oxidation, particularly in 1 M H_2_SO_4_, is an effective approach for improving the functional performance of Ti-6Al-4V ELI alloy, making it a promising candidate for biomedical applications requiring enhanced corrosion resistance and surface stability.

## Data Availability

The original contributions presented in this study are included in the article. Further inquiries can be directed to the corresponding authors.
